# Broadband RCS Reduction by a Quaternionic Metasurface

**DOI:** 10.3390/ma14112787

**Published:** 2021-05-24

**Authors:** Zhao Zhang, Yazhong Zhang, Tianlong Wu, Shaowen Chen, Wei Li, Jianguo Guan

**Affiliations:** 1 The First Aircraft Institute of AVIC, Xi’an 710089, China; hylonzz@sina.com; 2 State Key Laboratory of Advanced Technology for Materials Synthesis and Processing, International School of Materials Science and Engineering, Wuhan University of Technology, Wuhan 430070, China; yuezheng@whut.edu.cn (Y.Z.); tlwuoinc@whut.edu.cn (T.W.); chen_shaowen@whut.edu.cn (S.C.); guanjg@whut.edu.cn (J.G.)

**Keywords:** metasurface, absorbing material, metamaterial, RCS reduction

## Abstract

A quaternionic metasurface consisting of two pairs of units with destructive phase difference is proposed to extend the bandwidth of radar cross section (RCS) reduction. The two pairs of units are designed to have complementary phase-different bandwidth, which extends the bandwidth of RCS reduction. The overlaps of their bandwidth enhance the RCS reduction, resulting in a metasurface having broadband and strong RCS reduction. This design and the wideband RCS reduction of the quaternionic metasurface were verified by analytical calculation with superposition principle of electric field, numerical simulation with commercial software package CST Microwave Studio and experiment in microwave anechoic chamber. The scattering mechanism and the angular performance of the quaternionic metasurface were also investigated.

## 1. Introduction

In recent years, metamaterials attract extensive research interests because of their brand-new electromagnetic mechanism, designable material parameters and powerful ability in controlling reflection, propagation, and absorption of electromagnetic waves [1,2,3,4]. Metasurfaces are two-dimensional metamaterials which consist of sub-wavelength unit cells arranged in a plane in a periodical or deliberately disordered manner [5], offering extra advantages of low profile when inheriting the flexible design and outstanding performance of metamaterials [6]. In view of the advantages of the metamaterials/metasurfaces and the importance of microwave absorbing materials in both military and civilian applications [7,8], they are soon crossed over as metamaterial absorbers [1,9,10,11,12,13] or radar cross section (RCS) reducers [14,15], and become an important and prospective research field.

Metamaterial absorbers usually show narrow working bandwidth that is not favored for many practical applications [16,17]. Numerous efforts have been made to extend the bandwidth of metamaterial absorbers, typically by integrating multiple unit cells in plane or thickness didrection [18,19], usually resulted in multiple absorption peaks or even a broadband absorption bandwidth. The pyramidal metamaterials, which are constructed by stacking tens of layers of size gradient resonant unit cells, can realize an absorption bandwidth comparable to that of the best-performing 1 mm thick traditional magnetic absorbers in the range of 8–18 GHz [20,21,22]. Different from metamaterial absorbers which directly reduce the amplitude of microwaves by absorption, metasurfaces exploit the phase modulation of gradient changing unit cells to reduce backscattering by interference [23,24]. Based on this idea, the unit cells of metasurfaces with different phases are coded, designed, and programmed to show highly designable scattering patterns, including the ones that show extremely low radar observability [25,26,27,28]. The RCS reduction metasurfaces makes use of phase difference of neighboring unit cells other than the strong resonant absorption, granting them much broader working bandwidth and lower profile with respect to metamaterial absorbers [29,30]. For example, Cui et al. proposed a metasurface that composed of different square patches with a thickness of only 1.985 mm to exhibit a −10 dB RCS reduction in the frequency range of 7.8–12 GHz [31]. To simplify the design and fabrication of these metasurfaces, they are usually constructed by a pair of unit cells with opposed phases to form a chessboard-like arrangement. The frequency range of effective phase difference is closely related to the thickness of the dielectric layer, the frequency band of effective phase difference is limited for a certain thickness of the dielectric layer. Therefore, it is difficult to generate effective phase difference in the wideband by only a pair of unit cells.

In this work, a quaternionic metasurface with two pairs of anti-phase unit cells is proposed to achieve broadband RCS reduction. The two pairs of unit cells are designed to have opposed reflection phase in different frequency range. Consequently, the combination of them shows extended working bandwidth with respect to existing binary schemes that involves only one pair of anti-phase unit cells. The final RCS reduction bandwidth of −10 dB is from 13 to 32 GHz, which is verified by experimental measurements.

## 2. Design Principle of Metasurface

With the normal incidence of electromagnetic waves, the monostatic RCS reduction by a metasurface consisting with M × N units can be expressed as:

(1) $$RCSR_{normal}=20\log\left|\sum_{m=1}^{M}\sum_{N=1}^{N}E_{mn}e^{ik\beta_{mn}}\right|$$

where *E_mn_* and *β_mn_* represent the amplitude and phase of the reflected electric field of the unit in row *m* and column *n* of metasurface, respectively, and *k* is the wave vector of the incident electromagnetic wave in vacuum. The RCS reduction of the metasurface can be calculated by substituting the reflected amplitude and phase of all units into Equation (1). From Equation (1) we can also obtain the RCS reduction materials with the versatile and controllable properties by using different units. However, multifarious types of units will also complicate the design strategy and technical process. Consequently, the types of unit cell should be as less as possible in the premise of meeting the aimed requirements. In the simplest case, the RCS reduction formula of the chessboard metasurface with only two kinds of unit cells can be significantly simplified as:

(2) $$RCSR=20\log\left|\frac{A_{1}e^{ik\beta_{1}}+A_{2}e^{ik\beta_{2}}}{2}\right|$$

where *A* represents the amplitude of the reflected electromagnetic waves and the numeric subscripts correspond to different type of units. According to Equation (2), the RCS reduction can be achieved to some extent if the phase difference between unit ranges within 180 ± 60°, and −10 dB RCS reduction can be achieved when the phase difference ranges within 180 ± 37°. The RCS reduction formula for the metasurface, which is composed of two pairs of units (four types of units) can be described as:

(3) $$RCSR_{4units}=20\log\left|\frac{A_{1}e^{ik\beta_{+}}+A_{1}e^{ik\beta_{-}}+A_{2}e^{ik\beta_{+}}+A_{2}e^{ik\beta_{-}}}{4}\right|$$

the subscripts 1 and 2 represent the index of the paired metamaterial units, and *β_+_* and *β_−_* represent metamaterial units with positive and negative phases. According to Equation (3), the RCS reduction below −10 dB can be achieved as we obtain the same reflection amplitudes and the phase difference ranges within 180° ± 37° at the same time. One can also realize a good RCS reduction and the expansion bandwidth if only one pair of phase difference meets the 180° ± 37° condition and the other pair meets the condition of 180° ± 60°. Since the working band and reflection characteristics of the two pairs of cells can be designed separately, it can be expected that the quaternionic design possesses better wideband performance compared to the existing set of binary designs.

## 3. Results and Discussion

### 3.1. Anti-Phase Unit Cells

**Figure 1:**
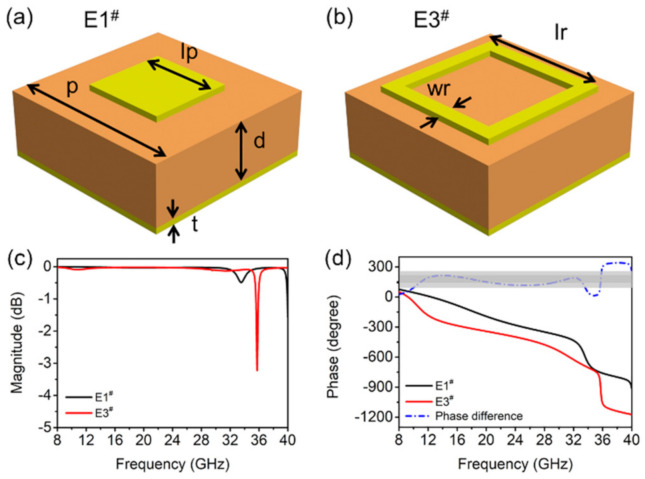
Schematic of the two units of (**a**) square patch and (**b**) square ring. The magnitude (**c**) and phase (**d**) of reflectivity of the two units.

The structural schematics of metamaterial units with the basic shapes are square plates and square rings, labeled as E1^#^ and E3^#^, as shown in Figure 1a,b. The pitch of both structural units is *p* = 6 mm, and other geometric parameters are *lp* = 2.8 mm, *lr* = 4.5 mm, and *wr* = 0.5 mm. The thickness of metal structures with the conductivity of 5.8 × 10^7^ S/m and dielectric spacer F4B plate with the complex permittivity of *ε_r_* = 2.65(1 − 0.001*i*) are *t* = 0.035 mm and *d* = 2 mm, respectively.

The reflection coefficient, including amplitude and phase of the electromagnetic wave of the above two units were simulated and presented in Figure 1c,d. From Figure 1c, E1^#^ and E3^#^ present a narrow band of weak absorption peaks at 33.6 GHz and 35.7 GHz, but show strong reflection to the incident electromagnetic wave in other frequency ranges (the reflection coefficient is close to 1). Figure 1d illustrates the phase difference between E1^#^ and E3^#^ is 180° ± 60° ranges within 9–34 GHz, which is conducive to RCS reduction. The phase difference between E1^#^ and E3^#^ is 180° ± 37° in the range of 10.9–20.2 GHz and 29.2–33.1 GHz, which meet the condition of −10 dB RCS reduction.

**Figure 2:**
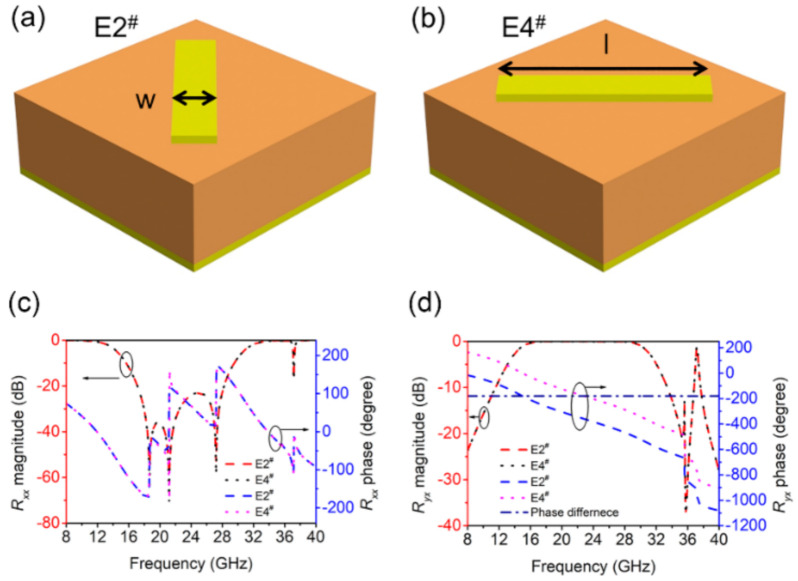
Schematic for the two units of (**a**) 45° cut-wire (E2^#^) and (**b**) 135° cut-wire (E4^#^). The magnitude and phase of (**c**) *R_xx_* and (**d**) *R_yx_* for E2^#^ and E4^#^.

As shown in Figure 2a,b, the structural units with cut-wire patterns arranged at 45° and 135° were designed, denoted as E2^#^ and E4^#^, where *l* = 5 mm and *w* = 1 mm. As the two basic units E2^#^ and E4^#^ are asymmetrical on the X and Y axes, their reflected waves produce polarization conversion. The amplitude and phase of the reflected electromagnetic waves are shown in Figure 2c,d. The co-polarized coefficient and the cross-polarized coefficient is defined as *R_xx_* = |*E_xr_*|/|*E_xi_*| and *R_yx_* = |*E_yr_*|/|*E_xi_*|, respectively. *R_xx_* and *R_yx_* denote the reflection ratio of *x*-to-*x* and *x*-to-*y* polarization conversion, respectively. From Figure 2c that the *R_xx_* (co-polarized coefficient) value of E2^#^ and E4^#^ are the same, and the reflectivity is low ranges within 16–29.2 GHz. Figure 2d shows, however, the value of *R_yx_* (cross-polarized coefficient) is close to 0 dB ranges within 16–29.2 GHz, which means an almost total reflection. From the results in Figure 2c,d, the two units of E2^#^ and E4^#^ only exhibit orthogonal polarization reflection in this frequency range. Figure 2d also shows that the *R_yx_* phase difference of E2^#^ and E4^#^ is always 180°, which means the same amplitude and opposite phase, so the condition of −10 dB RCS reduction is satisfied in the range of 16–29.2 GHz. Therefore, the cut-wire patterns arranged at 45° and 135° can achieve 180° phase difference in the maximum frequency range, as shown in Figure 2d.

### 3.2. Binary Metasurface

**Figure 3:**
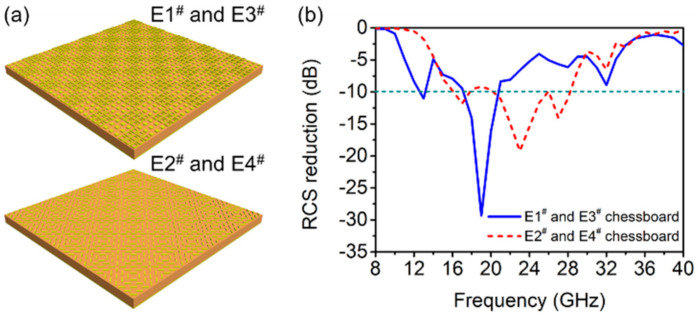
(**a**) The schematics of E1^#^, E3^#^ and E2^#^, E4^#^ chessboard metasurface. (**b**) Simulation result of the RCS reduction of the two chessboard metasurface.

As reported by many existing works, a pair of anti-phase unit cells can construct an RCS reduction metasurface that works in their anti-phase band. The above discussed anti-phase pairs, E1^#^, E3^#^ and E2^#^, E4^#^ are respectively arranged in chessboard-like manner, as shown in Figure 3a. With normal incidence of electromagnetic waves, the RCS reduction of the chessboard-like metasurfaces are simulated as in Figure 3b. The chessboard metasurface made of E1^#^ and E3^#^ shows three strong peaks of RCS reduction at 13 GHz, 19 GHz and 32 GHz, and the maximum reduction reach −30 dB at 19 GHz. The frequency band with RCS reduction of the metasurface is consistent with the frequency band with 180° phase difference between E1^#^ and E3^#^ units by comparing and analyzing Figure 1d and Figure 3b, which indicate that the RCS reduction is mainly due to the opposite phase of the reflected electromagnetic wave, leading to scattering caused by the interference in the far field. The RCS reduction of the metasurface can reach below −5 dB within 9–34 GHz, which is almost consistent with the phase difference of 180° ± 60°.

The RCS reduction of chessboard metasurface composed of E2^#^ and E4^#^ can achieve −10 dB within 16–29 GHz, among which three peaks of the RCS reduction occurs at 17 GHz, 23 GHz, and 27 GHz. Figure 2d and Figure 3b show that the bandwidth of the RCS reduction is consistent with the frequency of the total reflected cross-polarized electromagnetic wave of E2^#^ and E4^#^ units, which further confirms that the RCS reduction of the chessboard metasurface composed of E2^#^ and E4^#^ is owing to the scattering formed by the interference of the cross-polarized reflection waves of the two units ranges within 16–29 GHz.

From the above analysis, the working bandwidth of the RCS reduction of E1^#^, E3^#^ and E2^#^, E4^#^ chessboard metasurface are complementary to each other, which is because that the two pairs of metamaterial units with phase difference of 180° ± 37° are complementary. On this basis, the two pair of unit cells can be used to construct a quaternionic metasurface to achieve broadened bandwidth of RCS reduction.

### 3.3. Quaternionic Metasurface

**Figure 4:**
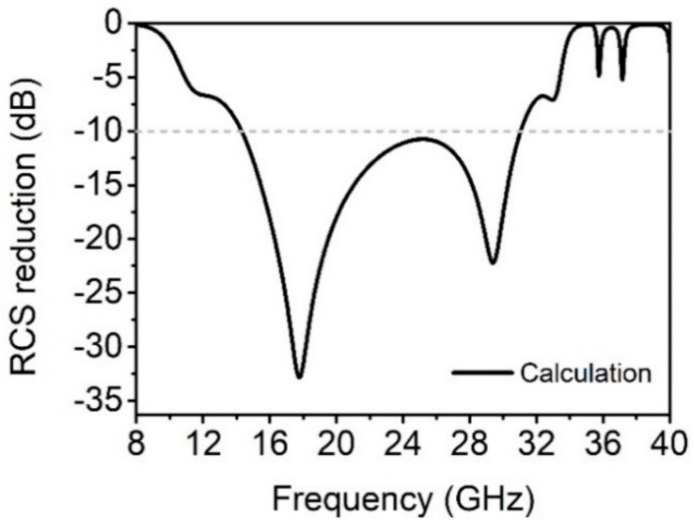
The calculated RCS reduction of the quaternionic metasurface based on Equation (3).

According to the design principle of RCS reduction above, the value of RCS reduction of the quaternionic metasurface can be calculated by substituting the reflection amplitude and phase values of E1^#^ to E4^#^ into Equation (3), as shown in Figure 4. The theoretical calculation results show that the quaternionic metasurface can achieve less than −10 dB RCS reduction in the broadband range of 14–32 GHz, and the value of the RCS reduction can reach −34 dB and −23 dB at 18 GHz and 30 GHz, respectively. On the other hand, the weak loss caused by the resonance absorption of the metamaterial unit itself was observed almost 36 GHz, which has little effect on the RCS reduction characteristics of the metasurface.

**Figure 5:**
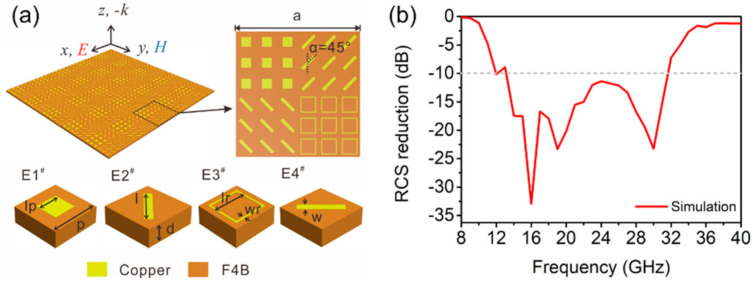
(**a**) The schematic of the quaternionic metasurface, and (**b**) simulation result of the RCS reduction.

The accuracy of the theoretical design and calculation results of the proposed metasurface was further verified by electromagnetic simulation. The schematic of the quaternionic metasurface, as shown in Figure 5a, where *a* = 36 mm and the other parameters are the same as the previous unit structure. The simulation results of RCS reduction of the quaternionic metasurface are illustrated in Figure 5b, which suggest the frequency range of the RCS reduction below −10 dB is 14–32 GHz. The simulation results are in good accordance with the theoretical calculation, validating the correctness of the design principle. More importantly, the −10 dB RCS reduction bandwidth is greatly expanded compared to the performance of the previous binary metasurfaces. The period of a single element in the quaternionic metasurface will affect the bandwidth of RCS reduction. When the period and structure size are reduced, the working frequency band will move to high frequency, and vice versa. If only the area of the full metasurface is enlarged by using different number of periodic elements, its working frequency band will not change.

**Figure 6:**
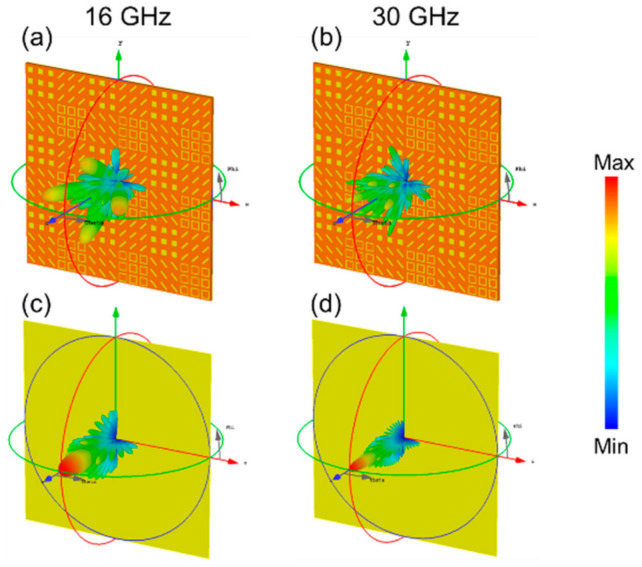
3D bistatic RCS patterns under normally incident waves for the quaternionic metasurface at (**a**) 16 GHz, (**b**) 30 GHz and the metal plate at (**c**) 16 GHz, (**d**) 30 GHz.

Figure 6 shows the simulated 3D far-field RCS patterns of the quaternionic metasurface and metal plate at 16 GHz and 30 GHz, respectively. At low frequency, the incident wave is mainly scattered into four strong lobes in the *xoz* and *yoz* planes and accompanied by some weak lobes. Since most of the electromagnetic energy is scattered to other directions rather than mirror reflection, backward RCS reduction takes effect. From Figure 6b,d, at high frequency, the perpendicular incident electromagnetic wave is also scattered into many beam lobes, different from the case when the electromagnetic wave incident on the metal plate. Meanwhile, the scattered beam gradually approaches to the center of *θ* = 0° and the scattering phenomenon weakens with the increase of the frequency.

**Figure 7:**
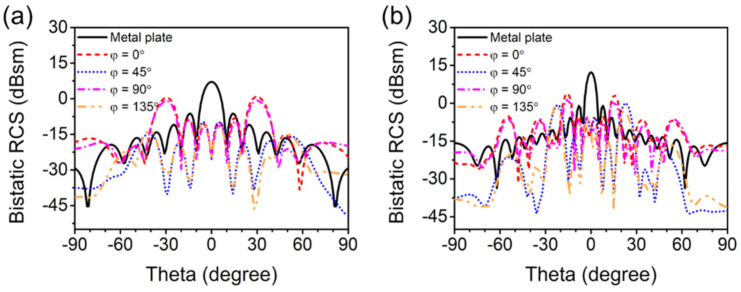
Bistatic RCS versus elevation angle (*θ*) for *φ* = 0°, 45°, 90° and 135° cut-planes under normal incidence at (**a**) 16 GHz and (**b**) 30 GHz.

Figure 7 shows the simulation results of 2D bistatic RCS of the quaternionic metasurface at 16 GHz and 30 GHz, which can be used to quantitatively analyze the effect of RCS reduction of the metasurface. At 16 GHz, the strongest reflected beam of the metal plate occurs at *θ* = 0° and its intensity is 7.12 dBsm, while the strongest of the metasurface reflected waves are mainly scattered into four beams at (*φ* = 0° *θ* = ±30°) and (*φ* = 90° *θ* = ±30°) and the strength reduced by around 7 dBsm. Moreover, the metasurface shows more strong scattering beams at 30 GHz, including those at *φ* = 0°, 45°, 90° and 135°. In the case of Figure 7b, the angle of the scattering beam is the same when φ = 0° and 90°, mainly concentrated in *θ* = ±15°, 35° and 55°. Nonetheless, when *φ* = 45° and 135°, the scattered beams increase and disperse, mainly concentrated below θ = 30°, and two stronger of the side lobes can be observed at *θ* = ±22°. At this frequency, the maximum RCS of the metasurface is about 8.45 dBsm lower than that of the metal plate. Therefore, it can be confirmed that the scattering effect of the metasurface can effectively reduce the backward RCS of the object.

### 3.4. Fabrication and Measurement

**Figure 8:**
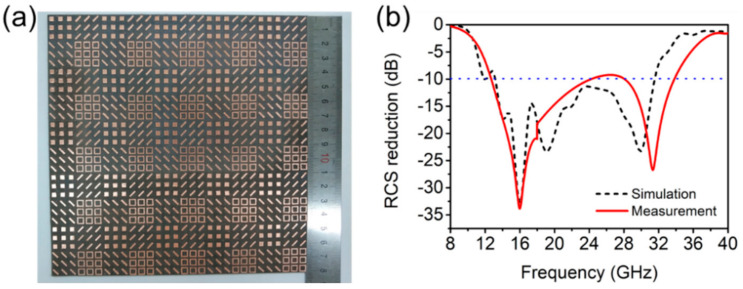
(**a**) Photograph of the fabricated sample, and its (**b**) simulated and measured RCS reduction.

The sample of the quaternionic metasurface with a dimension of 180 mm × 180 mm × 2 mm were fabricated by traditional PCB technology [32]. In Figure 1a, it is composed of 5 × 5 large units arranged periodically. The reflectivity of the fabricated metasurface and metal plate was measured through the NRL-arch method [33], and then was converted into RCS reduction, as shown in Figure 8b. The figure indicates that the bandwidth of −10 dB RCS reduction is 12.7–33.8 GHz, and the relative bandwidth is up to 90%. Small deviations between the measured and the simulated results can be observed, especially at near 30 GHz, which is mainly due to the fact that the material parameters of the dielectric plate used are not absolutely consistent with the parameters set by the simulation and the size of the actual sample is slightly different from that of the simulation model. Besides, the simulation curve is drawn by a limited number of points, which is not smooth enough, while the actual measured curve is obtained by hundreds of frequency data. However, the measured results of the metasurface are in good accordance with the simulation results in other frequency ranges, which illuminates that the designed quaternionic metasurface with good RCS reduction effect in the broadband range can be achieved.

## 4. Conclusions

Complementary phase difference wavebands is achieved by a quaternionic metasurface which is composed of two pairs of metasurface unit cells. By combining the two pairs of unit cells with effective phase difference (180 ± 60°) in different frequency ranges, the bandwidth of radar cross section (RCS) reduction is greatly expanded, while the effective phase difference in the same broadband enhances the RCS reduction. Theoretical calculations, simulations and experiments verified that the less than −10 dB RCS reduction can be achieved in the broadband of 13–33 GHz, and the RCS reduction is mainly achieved by controlling the angular distribution of electromagnetic wave energy rather than absorption. This work provides an effective method for the design of wideband RCS reducer that is important for radar stealth, anechoic chamber and electromagnetic compatibility.

## Data Availability

Data sharing not applicable.
